# CN-VEFD: A visual emotion feature dataset for Chinese ESG reports

**DOI:** 10.1016/j.dib.2025.112305

**Published:** 2025-11-22

**Authors:** Zhao Duan, Yitong Xu, Binglong Xia

**Affiliations:** School of Information Management, Central China Normal University, Wuhan 430079, China

**Keywords:** Color harmony, Layout aesthetics, Saliency detection, Facial expression recognition, Image analytics, Sustainability disclosure, Stakeholder perception

## Abstract

As ESG reporting gains prominence, visual elements play a critical role in shaping stakeholder perceptions, yet most research prioritizes textual analysis and overlooks visual emotion. We present CN-VEFD, a large-scale visual emotion feature dataset derived from 13,481 ESG reports by Chinese listed companies spanning 2006–2023. The dataset contains 59 structured visual emotion features computed from 399,321 images, covering three dimensions: color emotion, composition emotion, and facial emotion. In addition, 7 basic information fields are provided to link images to reports and firms (total 66 fields). Features are quantified through an automated pipeline that integrates color analysis, saliency detection, and facial emotion recognition. CN-VEFD supports research in visual analytics, emotion recognition, and behavioral finance, enabling systematic exploration of how visual emotional signals in ESG disclosures relate to corporate governance and stakeholder responses.

Specifications Table

**Table:** 

Subject	Computer Sciences
Specific subject area	Visual analytics, visual emotion recognition, ESG reporting analysis
Type of data	Table, Processed.
Data collection	ESG reports (2006–2023) from Chinese listed firms collected via stock exchanges (SSE, SZSE) and databases (CSMAR, RESSET); 399,321 images extracted from 13,481 PDF reports; 59 visual emotion features (color, composition, facial) computed via an automated pipeline integrating color analysis, saliency detection, and facial emotion recognition, plus 7 basic information fields; features validated on a sample subset.
Data source location	Institution: Central China Normal University, Wuhan 430,079, China Source of the primary data: CSMAR (Website: https://data.csmar.com) RESSET (Website: https://www.resset.com) CNINFO Data Service (Website: https://www.cninfo.com.cn) Shanghai Stock Exchange (Website: http://www.sse.com.cn/) Shenzhen Stock Exchange (Website: http://www.szse.cn/)
Data accessibility	Repository name: Mendeley Data Data identification number: 10.17632/3x9654y7fm.2 Direct URL to data: http://www.szse.cn/https://data.mendeley.com/datasets/3x9654y7fm/2
Related research article	none

## 1 Value of the Data

• These data provide 59 structured visual emotion features extracted from 399,321 images across 13,481 ESG reports (2006–2023) from Chinese listed companies, enabling large-scale empirical research on visual communication strategies in corporate sustainability disclosure.
• Researchers in behavioral finance, corporate governance, and visual analytics can leverage these data to investigate relationships between visual presentation choices (color harmony, compositional balance, facial expressions) and corporate outcomes (ESG ratings, stock market reactions, stakeholder engagement).
• The dataset facilitates development and benchmarking of visual emotion recognition models, multi-modal analysis algorithms, and automated ESG report assessment tools, advancing computational methods in document image analysis.
• Quantitative visual emotion metrics enable systematic comparison across industries, time periods, and company characteristics, supporting evidence-based recommendations for corporate communication strategies and regulatory policy development.

## 2 Background

As the concept of ESG (Environmental, Social, and Governance) becomes increasingly important in corporate information disclosure and capital markets, the visual elements (images, charts, layouts, and color schemes) in ESG reports not only serve an information transmission function but may also influence stakeholders’ emotional perceptions and decision-making judgments. However, existing research has primarily focused on the textual readability of ESG reports [1,2], textual attributes [3], and textual sentiment analysis [4,5], with relatively little research on the emotional communication effects of visual elements in reports and their quantitative measurement. The CN-VEFD dataset provides a large-scale resource for exploring the potential connections between visual presentation and corporate governance, as well as the effectiveness of information disclosure.

## 3 Data Description

The CN-VEFD dataset provides a multi-dimensional visual emotion annotation system. The primary data file CN-VEFD.csv contains 59 visual emotion features + 7 basic information fields (total 66) organized into four categories. The first category is Basic Information, which includes 7 fields: Year, stock code, page number, company name (Chinese/English), and industry classification (Chinese/English). The second category is Color Emotion Features, comprising 17 fields such as dominant color (RGB values and emotion mapping), positive/negative/neutral color ratios, emotion intensity score, rhythm score, color distribution uniformity, dominant hues, harmony score, and angular difference. The third category is Compositional Features, containing 20 fields including visual centroid coordinates, left-right and top-bottom balance scores, edge density, line counts (horizontal/vertical/diagonal), circle detection metrics, element count, whitespace ratio, element density and spacing, focus position and strength, and aspect ratio. The fourth category is Facial Emotion Features, with 22 fields covering face count, face area ratio, dominant facial emotion category and confidence, emotion intensity scores (positive/negative/neutral), emotion polarity and diversity, and probabilities for eight emotion categories (neutral, happiness, sadness, surprise, anger, contempt, fear, disgust). For clarity, “features” refer to the 59 visual emotion features, while “fields” denote all 66 columns (59 + 7).

Repository contents:

- CN-VEFD.csv: full feature table with 59 visual emotion features + 7 basic information fields (total 66; see Table 3).
- code/: scripts to reproduce feature extraction
- color_emotion.py; composition_emotion.py; face_emotion.py; main.py
- esg/: helper module (esg.py) with placeholder paths to be configured before running

Note on raw images: original report PDFs and extracted images are not redistributed; only derived, non-identifiable feature tables are shared.

Table 1 reports the distribution of reports and images by industry. The manufacturing sector has the largest representation with 1412 companies and 7448 observations, followed by the finance sector with 969 observations and information technology services with 744 observations.

**Table 1:** Distribution of reports and images by industry.

Industry	Companies	Year Span	Observations
Mining	59	16	469
Electricity, Heat, Gas and Water Production and Supply	93	18	646
Real Estate	65	17	559
Construction	54	16	326
Transportation, Storage and Postal Services	78	18	606
Education	4	6	15
Finance	115	17	969
Scientific Research and Technical Services	45	11	99
Agriculture, Forestry, Animal Husbandry and Fishery	27	15	152
Wholesale and Retail Trade	94	17	631
Water Conservancy, Environment and Public Facilities Management	45	16	158
Health and Social Work	13	17	68
Culture, Sports and Entertainment	44	17	285
Information Transmission, Software and Information Technology Services	134	18	744
Manufacturing	1412	18	7448
Accommodation and Catering	29	17	86
Comprehensive	5	16	38
Leasing and Business Services	41	18	182
Total	2357	18	13,481

Note: “Year Span” denotes the number of distinct years covered within each industry (2006–2023) and is not additive across industries.

Table 2 reports the temporal distribution of the dataset from 2006 to 2023. In 2006, there were 16 reports contributing 212 images, averaging 13.3 images per report. By 2023, the dataset expanded to 2166 reports with 98,344 images, averaging 45.4 images per report, demonstrating significant growth in ESG reporting practices over time.

**Table 2:** Distribution of reports and images by year.

Year	Number of Reports	Number of Images	Images per Report
2006	16	212	13.3
2007	40	439	11.0
2008	177	2104	11.9
2009	184	2962	16.1
2010	492	9044	18.4
2011	588	11,875	20.2
2012	653	14,607	22.4
2013	697	16,361	23.5
2014	715	16,840	23.6
2015	767	18,675	24.3
2016	805	20,494	25.5
2017	844	21,599	25.6
2018	898	23,883	26.6
2019	933	26,655	28.6
2020	1047	32,173	30.7
2021	1298	44,670	34.4
2022	1161	38,384	33.1
2023	2166	98,344	45.4
Total	13,481	399,321	29.6

Table 3 provides a detailed list of all 59 visual emotion features + 7 basic information fields (total 66) in the dataset, including variable names, descriptions, and definitions for each feature across the four categories.

**Table 3:** Data Structure.

	Variable	Description
1	year	Year
2	code	Stock Code
3	page	Page Number
4	company_name_zh	Company Name (Chinese)
5	company_name_en	Company Name (English)
6	industry_zh	Industry Classification (Chinese)
7	industry_en	Industry Classification (English)
8	dominant_color	Dominant Color Name
9	dominant_color_rgb_red	Dominant Color R Component (0–255)
10	dominant_color_rgb_green	Dominant Color G Component (0–255)
11	dominant_color_rgb_blue	Dominant Color B Component (0–255)
12	dominant_color_emotion	Emotion Description Mapped from Dominant Color
13	dominant_color_category	Emotion Category (Positive/Negative/Neutral)
14	positive_ratio	Ratio of Foreground Pixels Classified as Positive Colors
15	negative_ratio	Ratio of Foreground Pixels Classified as Negative Colors
16	neutral_ratio	Ratio of Foreground Pixels Classified as Neutral Colors
17	emotion_intensity_score	Color Emotion Intensity Score (0–1)
18	rhythm_score	Color Rhythm Raw Score Based on Color Transitions
19	emotion_rhythm_score	Emotion Rhythm Score (Normalized)
20	color_distribution_uniformity	Color Distribution Uniformity Index
21	dominant_hues_1	First Dominant Hue (HSV Hue Value, 0–180)
22	dominant_hues_2	Second Dominant Hue (HSV Hue Value, 0–180)
23	harmony_score	Hue Harmony Score Based on Color Wheel Theory
24	angle_diff	Angular Difference Between Dominant Hues (0–180°)
25	visual_centroid_x	Visual Centroid X Coordinate (Saliency-weighted)
26	visual_centroid_y	Visual Centroid Y Coordinate (Saliency-weighted)
27	balance_lr	Left-Right Visual Balance Score (0–1)
28	balance_tb	Top-Bottom Visual Balance Score (0–1)
29	edge_density	Edge Density via Canny Edge Detection (0–1)
30	line_count	Number of Lines Detected via Hough Transform
31	horizontal_line_ratio	Proportion of Horizontal Lines (0–1)
32	vertical_line_ratio	Proportion of Vertical Lines (0–1)
33	diagonal_line_ratio	Proportion of Diagonal Lines (0–1)
34	circle_count	Number of Circles Detected via Hough Circle Transform
35	circle_area_ratio	Total Circle Area to Image Area Ratio
36	elements_count	Number of Salient Elements (Connected Components)
37	whitespace_ratio	Whitespace Ratio Based on Saliency Thresholding (01)
38	element_density	Salient Elements Density per Unit Area
39	mean_element_distance	Mean Euclidean Distance Between Element Centroids
40	mean_element_distance_norm	Normalized Mean Distance (Image Diagonal-based)
41	focus_position_x	Primary Focus X Position (Max Saliency Point)
42	focus_position_y	Primary Focus Y Position (Max Saliency Point)
43	focus_strength	Focus Strength (Max Saliency Value, 0–1)
44	aspect_ratio	Image Width to Height Ratio
45	face_count	Number of Faces Detected (MTCNN)
46	total_face_area_ratio	Total Face Area to Image Area Ratio
47	dominant_face_emotion	Dominant Facial Emotion Category (Area-weighted)
48	dominant_face_emotion_confidence	Average Confidence Score of Dominant Emotion
49	face_emotion_intensity_score	Composite Facial Emotion Intensity Score
50	positive_face_emotion_score	Area-weighted Positive Facial Emotion Score
51	negative_face_emotion_score	Area-weighted Negative Facial Emotion Score
52	neutral_face_emotion_score	Area-weighted Neutral Facial Emotion Score
53	face_emotion_polarity	Facial Emotion Polarity (Positive - Negative)
54	face_emotion_diversity	Shannon Entropy of Facial Emotion Distribution
55	face_emotion_category	Image-level Facial Emotion Category
56	neutral_prob	Area-weighted Neutral Emotion Probability
57	happiness_prob	Area-weighted Happiness Emotion Probability
58	sadness_prob	Area-weighted Sadness Emotion Probability
59	surprise_prob	Area-weighted Surprise Emotion Probability
60	anger_prob	Area-weighted Anger Emotion Probability
61	contempt_prob	Area-weighted Contempt Emotion Probability
62	fear_prob	Area-weighted Fear Emotion Probability
63	disgust_prob	Area-weighted Disgust Emotion Probability
64	face_emotion_confidence_min	Minimum Confidence Across All Detected Faces
65	face_emotion_confidence_max	Maximum Confidence Across All Detected Faces
66	face_emotion_confidence_std	Standard Deviation of Face Emotion Confidence

**Fig. 1:**
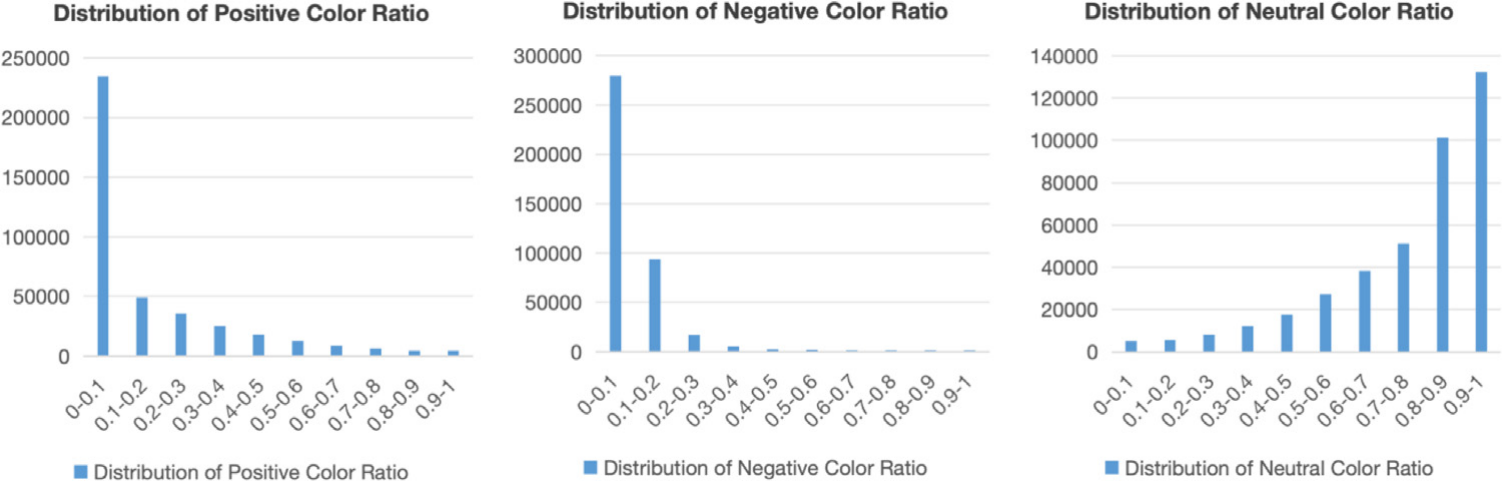
Distributions of Positive, Negative, and Neutral Color Ratios.

Fig. 1 displays the distributions of positive, negative, and neutral color ratios across the entire dataset, illustrating the emotional tone conveyed through color choices in ESG reports.

**Fig. 2:**
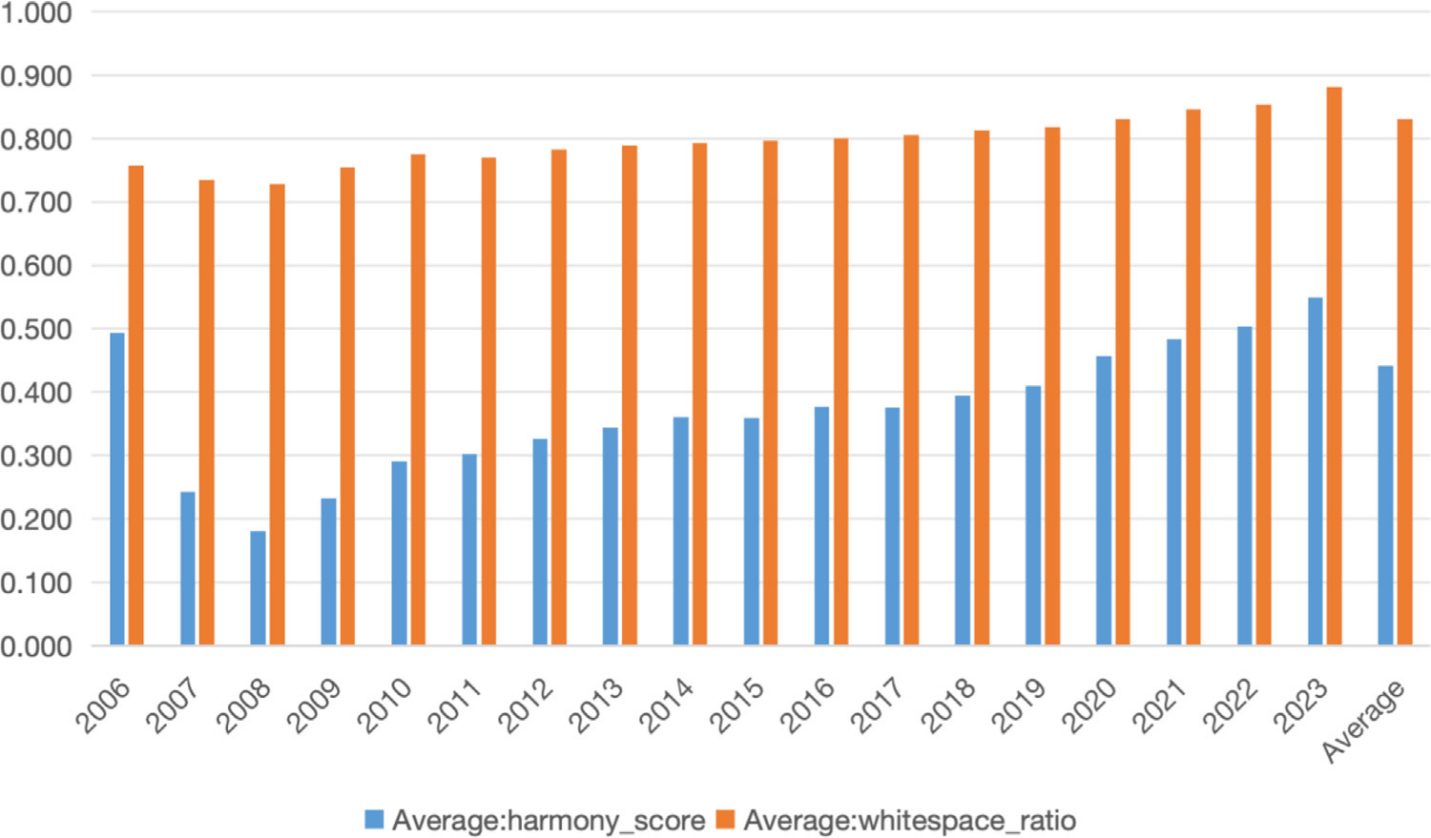
Annual trends of harmony score and whitespace ratio.

Fig. 2 displays the annual trends of hue harmony score and whitespace ratio from 2006 to 2023, revealing temporal patterns in visual design practices within Chinese ESG reporting.

## 4 Experimental Design, Materials and Methods

### 4.1 Data collection and preprocessing

ESG reports (2006–2023) from Chinese listed companies were collected from stock exchanges (SSE, SZSE) and databases (CSMAR, RESSET). Images were extracted from 13,481 PDF files using PyMuPDF (fitz 1.18.14), yielding 399,321 images. Data quality control included MD5-based deduplication, resolution filtering (≥50×50 pixels), and content review to remove duplicate, low-quality, or irrelevant images.

### 4.2 Computing environment

The automated pipeline was implemented in Python 3.10 on Ubuntu 20.04 LTS with Intel Xeon E5–2680 v4 (28 cores, 2.4 GHz) and NVIDIA Tesla V100 GPUs (32GB). Core dependencies included OpenCV 4.5.3, scikit-image 0.18.3, scikit-learn 0.24.2, NumPy 1.21.2, pandas 1.3.3, TensorFlow 2.6.0, mtcnn 0.1.1, and XEdu.hub 0.2.0. All stochastic operations used fixed random seeds (random_state=42).

### 4.3 Feature extraction pipeline

A systematic three-module pipeline for visual emotion analysis was implemented: (i) a color-emotion module quantifying color characteristics via color-space conversion, dominant-color extraction, and emotion mapping to produce metrics such as positive, negative, and neutral color ratios; Emotion mapping is grounded in principles of color psychology: high-saturation warm hues (red, orange, yellow) are mapped to positive affect, low-saturation cool hues (blue, violet) to negative affect, and neutral colors (green, gray) are assigned neutral valence. The specific implementation is defined in the ‘COLOR_EMOTION_MAP’ dictionary*.* (ii) a compositional-emotion module assessing compositional stability using saliency detection, visual-balance computation, and geometric element analysis; (iii) a facial-emotion module applying face detection and emotion recognition models, aggregating results with an area-weighted strategy to derive overall emotion distributions. Certain continuous features were normalized to the [0, 1] interval using Min-Max scaling based on their empirical distributions across the full dataset, while count-based features were retained in their original integer form. A subset of samples was manually validated to ensure annotation quality.

### 4.4 Color emotion analysis

Color carries significant meaning and can profoundly impact people’s emotions, cognition, and behavior.In visual emotion analysis, color is a key visual feature that strongly influences the emotional perception of images [6]. Drawing on psychology and art theory, prior works by Machajdik [7] and Valdez [8] inform the extraction of color features—such as dominant colors, color intensity, and color rhythm—that are salient for emotion recognition. Siersdorfer [9] found that emotional polarity in Flickr metadata is strongly correlated with color features like RGB histograms. Machajdik [7] categorized eight emotions based on color features into positive, negative, and neutral categories.

**Fig. 3:**
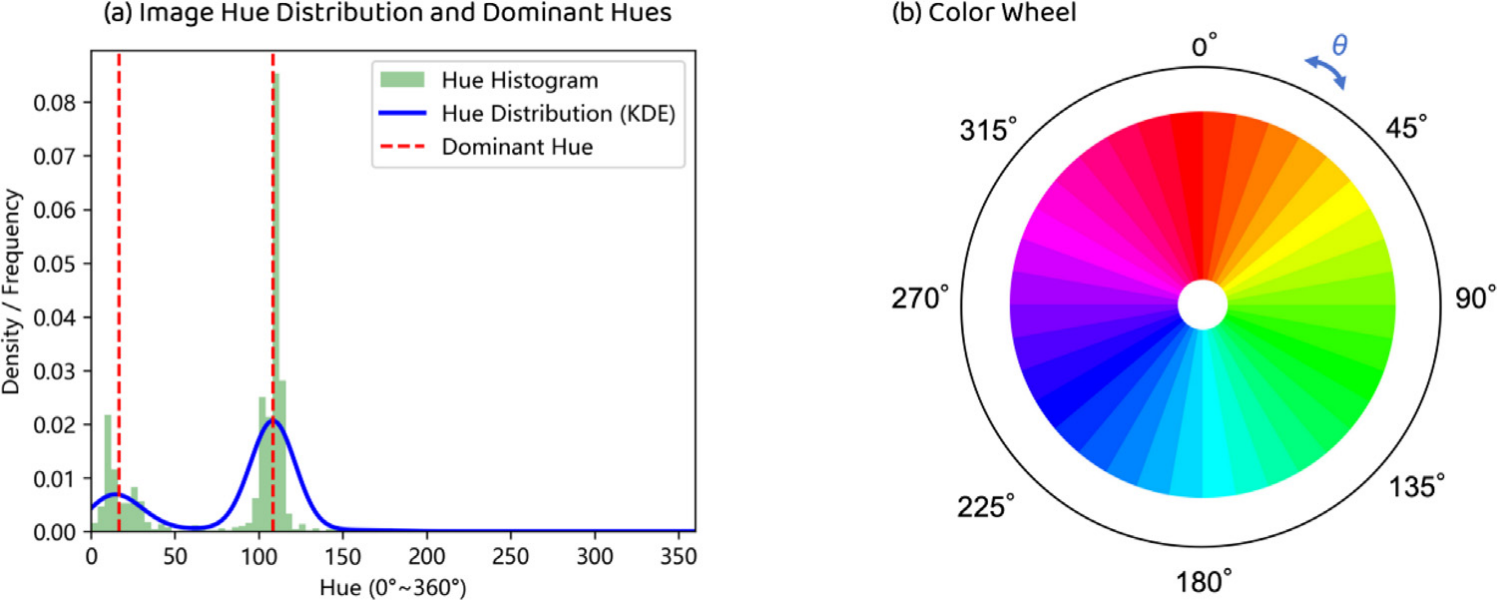
Detecting top two colors and measuring their internal angle (θ) on color wheel for color harmony.

Building on these findings, CN-VEFD classifies colors into emotional categories and computes their proportions within foreground pixels (positive/negative/neutral ratio) to quantify image-level color-emotion distributions. Harmonious color combinations can evoke positive responses. Fig. 3(a) Hue Distribution and Dominant Hues:The green bar chart represents the hue histogram, while the blue curve illustrates the continuous distribution of image hues. The two red dashed lines indicate the first and second dominant hues, respectively. Fig. 3(b) Color Wheel: On the standard hue circle, the angular separation θ (measured clockwise) between the two dominant hues serves as the core input for computing color harmony, thereby quantifying the aesthetic coherence of the color combination.Datta suggested that pairing two colors 180° apart on the color wheel produces the highest sense of harmony [10]. The pipeline implements an optimized version of Kim’s harmony calculation [11] by clustering to extract two dominant colors and applying hue-harmony analysis to assess the harmony of color combinations in images.

### 4.5 Compositional emotion analysis

In the visual communication of ESG reports, image emotions can be identified from both global and local perspectives using image semantics, aesthetics, and low-level visual features [12]. In graphic design, a visual hierarchy that integrates graphics, text, and color forms an aesthetically meaningful language that conveys emotion: ordered color schemes and repetitive shapes tend to elicit positive responses, whereas chaotic compositions may evoke negative feelings. Visual elements such as line direction and pattern complexity are directly linked to emotion (e.g., differently oriented colored lines can induce a sense of motion, and patterns with complex centers and simple edges align more closely with viewers’ aesthetic cognition, reducing emotion-recognition bias) [13]. Structures grounded in architectural principles—including grid layouts, the golden ratio, and symmetrical balance—underpin aesthetic formation. A multi-level compositional feature-extraction pipeline is applied: the Spectral Residual algorithm [14] generates saliency maps to locate visual foci and compute visual centroids and balance; Canny edge detection quantifies image complexity; the Hough transform detects linear and circular structures; and layout analysis on binarized saliency maps extracts compositional attributes such as element count, density, spacing, and whitespace ratio.

### 4.6 Facial emotion analysis

Facial expressions are a key nonverbal channel for conveying emotion in interpersonal communication. As a core carrier of emotional expression.they play an important role in the visual communication of ESG reports. Facial emotions can directly convey corporate culture and values and shape stakeholders’ perceptions and emotional tendencies toward the corporate image. Recent advances in facial expression recognition have significantly improved model robustness with respect to rotation invariance, pose variation, and occlusion handling [[15], [16], [17], [18]]. However, these general-purpose models exhibit certain limitations when applied to the context of ESG reporting: their emotion label systems are misaligned with corporate discourse, they demonstrate recognition bias toward professionalized facial expressions, and they lack effective mechanisms for aggregating emotional states across multiple faces. To address these limitations,An integrated facial emotion analysis framework is implemented, leveraging the MTCNN algorithm [19] and the FERplus dataset [20], and combining high-precision face detection with multi-class emotion recognition to enable automated identification and quantitative analysis of facial emotions in images. Using an area-weighted aggregation strategy, individual facial emotion outputs are converted into image-level emotion distributions—covering positive, negative, and neutral dimensions—and further aggregated into comprehensive indicators such as emotional polarity and diversity.

The entire data processing workflow is designed for batch processing, supporting automated analysis of large-scale images. Some numerical features are appropriately normalized to ensure comparability across different metric dimensions. An error handling mechanism ensures that the failure to process individual images does not affect the overall analysis process, while detailed log information is recorded for subsequent quality control and result verification.

**Fig. 4:**
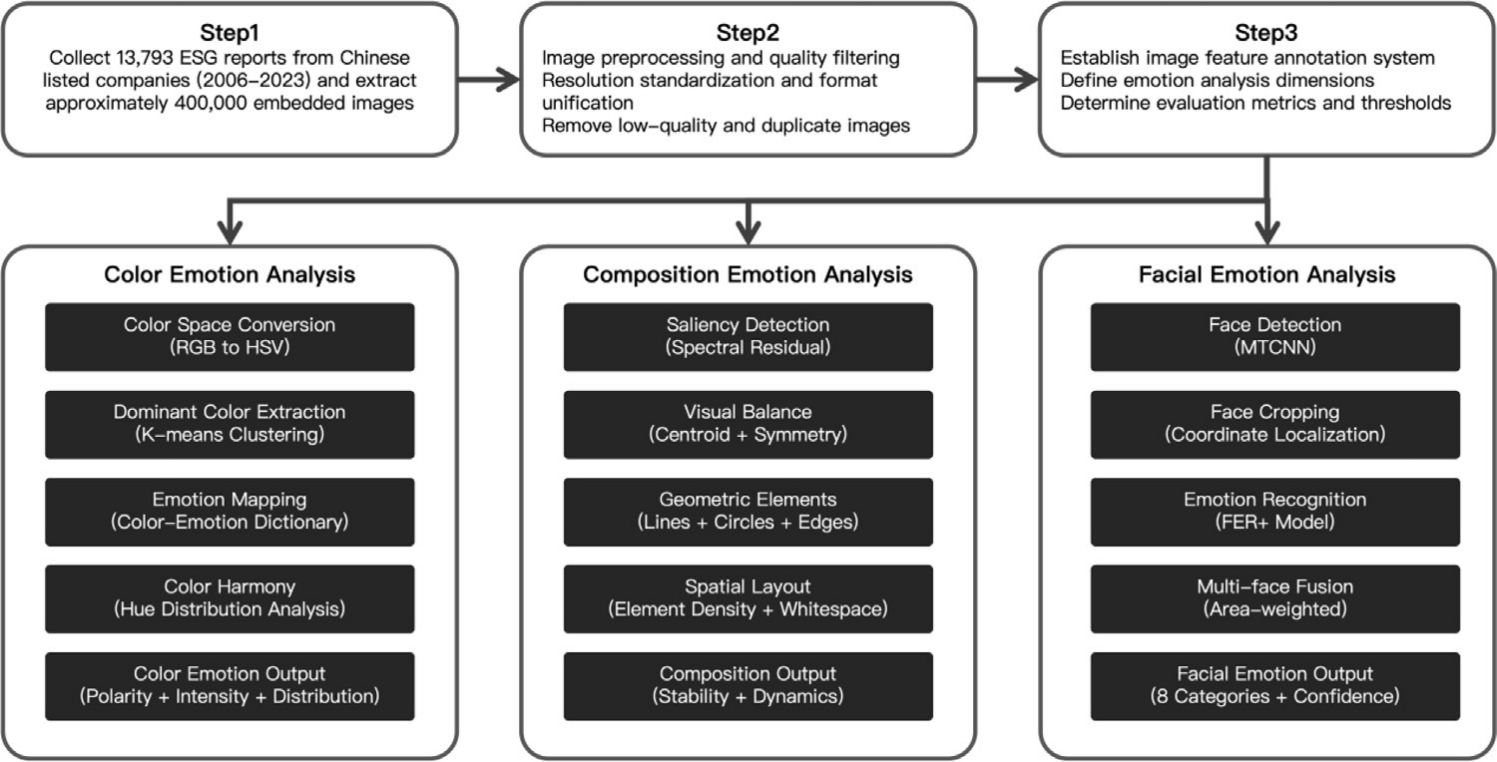
CN-VEFD dataset creation process.

The end-to-end dataset creation process is summarized in Fig. 4. (1) A corpus of 13,793 ESG reports published by Chinese listed companies between 2006 and 2023 was collected, from which approximately 400,000 embedded images were extracted.(2) An image feature annotation framework was established, accompanied by rigorous quality control procedures, including resolution standardization and removal of duplicate images.(3) A three-module sentiment analysis pipeline was implemented: color features were derived through color space transformation and clustering analysis; compositional features were analyzed via saliency detection and geometric element extraction; and a multi-face emotion recognition and fusion mechanism was realized using an area-weighted aggregation strategy.

### 4.7 Validation protocol

To evaluate the reliability of the automated feature extraction pipeline, we employed a stratified random sampling strategy to select a validation subset of 1500 images (approximately 0.39 % of the total dataset of 399,321 images). Stratification was conducted according to two criteria: publication year (proportional to the annual distribution of images from 2006 to 2023) and industry sector (covering the five largest sectors: manufacturing, finance, information technology, utilities, and real estate), ensuring representative coverage across temporal and sectoral dimensions.

Three trained annotators independently performed manual labeling. To minimize inter-annotator variability and subjectivity, consensus labels were established using a majority-rule protocol: for any given image-feature pair, if two or more annotators provided identical judgments, that judgment was adopted as the consensus ground truth. The outputs of the automated pipeline were then compared against this consensus label set, yielding an overall agreement rate of 95.6 %.

## Limitations

This dataset has several limitations: (1) Automated feature extraction may introduce recognition errors, particularly in facial emotion detection and saliency estimation; (2) The sample is limited to Chinese listed companies' ESG reports (2006–2023), restricting generalizability to other regions or private firms; (3) Manual validation covered only a subset due to scale constraints; (4) Temporal bias exists as early years (2006–2010) have sparse data; (5) Industry representation is uneven, with manufacturing dominating; (6) Dynamic visual elements (animations, videos) in digital reports are not captured. Future work can enhance this dataset along three principal directions:First, the integration of vision–language foundation models or domain-specific fine-tuning strategies could strengthen recognition robustness, while expanding the sample scope to include cross-cultural, cross-industry, and longitudinal instances would improve representativeness.Second, historical data gaps and sectoral biases may be mitigated through retrospective data mining, crowdsourced annotation, and industry partnerships. Furthermore, incorporating video frame analysis capabilities would enable the dataset to encompass dynamic, multimodal ESG disclosures.

## Ethics Statement

The authors have read and followed the ethical requirements for publication in Data in Brief. This work does not involve human subjects, animal experiments, or social media data. All data derive from publicly available corporate ESG reports.

## CRediT Author Statement

**Zhao Duan:** Supervision, Project administration, Funding acquisition, Writing – review & editing; **Yitong Xu:** Conceptualization, Methodology, Investigation, Data curation; **Binglong Xia:** Software, Formal analysis, Validation, Data curation, Writing – original draft, Visualization

## Data Availability

Mendeley DataCN-VEFD: A Visual Emotion Feature Dataset for Chinese ESG Reports (Original data)
